# Case Report: Rituximab Improved Epileptic Spasms and EEG Abnormalities in an Infant With West Syndrome and Anti-NMDAR Encephalitis Associated With APECED

**DOI:** 10.3389/fneur.2021.679164

**Published:** 2021-05-20

**Authors:** Go Kawano, Takaoki Yokochi, Ryuta Nishikomori, Yoriko Watanabe, Keizo Ohbu, Yukitoshi Takahashi, Haruo Shintaku, Toyojiro Matsuishi

**Affiliations:** ^1^Department of Paediatrics, St Mary's Hospital, Kurume, Fukuoka, Japan; ^2^Department of Paediatrics and Child Health, Kurume University School of Medicine, Kurume, Fukuoka, Japan; ^3^National Epilepsy Centre, National Hospital Organization (NHO) Shizuoka Institute of Epilepsy and Neurological Disorder, Shizuoka, Japan; ^4^Department of Paediatrics, Osaka City University Graduate School of Medicine, Osaka, Japan; ^5^Research Centre for Children and Research Centre for Rett Syndrome, St Mary's Hospital, Kurume, Fukuoka, Japan; ^6^Division of Gene Therapy and Regenerative Medicine, Cognitive and Molecular Research Institute of Brain Diseases, Kurume University School of Medicine, Kurume, Fukuoka, Japan

**Keywords:** autoimmune polyendocrinopathy-candidiasis-ectodermal dystrophy syndrome, infantile spasms, West syndrome, hypsarrhythmia, anti-N-methyl-D-aspartate receptor encephalitis, rituximab, epileptic spasms

## Abstract

**Background:** Autoimmune polyendocrinopathy-candidiasis-ectodermal dystrophy is a rare autosomal recessive disorder caused by a mutation in the autoimmune regulator gene. Patients with autoimmune polyendocrinopathy-candidiasis-ectodermal dystrophy typically exhibit hypoparathyroidism, adrenocortical failure, and chronic mucocutaneous candidiasis. There are only a few case reports of autoimmune encephalitis during autoimmune polyendocrinopathy-candidiasis-ectodermal dystrophy, but not as an initial manifestation. Furthermore, there are no reports of patients with infantile spasms/West syndrome with autoimmune encephalitis, partly because the median age for paediatric patients with anti-N-methyl-D-aspartate receptor encephalitis, which is the most frequent and best characterised in paediatric autoimmune encephalitides, is 13–14 years. Herein, we present a case of a 3-month-old infant with autoimmune encephalitis as an initial manifestation of autoimmune polyendocrinopathy-candidiasis-ectodermal dystrophy who later developed infantile spasms/West syndrome.

**Case Presentation:** A 3-month-old girl was admitted to our hospital with a fever, involuntary movements in all four limbs, and right-side facial palsy. Acute central nervous system demyelination diseases were suspected from neuroimaging findings and the presence of the cerebrospinal fluid oligoclonal band. She did not respond to multiple methylprednisolone pulse therapies and later developed infantile spasms/West syndrome and diabetes mellitus. Rituximab, a chimeric mouse/human monoclonal antibody directed against human CD20 which depletes B cells, was initially administered as a treatment for autoimmune encephalitis. Unexpectedly, this treatment resulted in complete spasm cessation and resolution of hypsarrhythmia. The patient eventually showed severely delayed developmental milestones, and her electroencephalography findings showed periodic generalised slow spike-and-wave pattern.

**Conclusions:** Despite the limited ability to extrapolate findings from a single case, rituximab's effects may suggest that B cells play a crucial role in infantile spasms/West syndrome mechanisms; use of rituximab as an aetiology-specific treatment for infantile spasms/West syndrome patients with autoimmune encephalitis or its effectiveness for infantile spasms/West syndrome patients with other underlying mechanisms warrants further investigation.

## Introduction

Autoimmune polyendocrinopathy-candidiasis-ectodermal dystrophy (APECED) syndrome, also known as autoimmune polyendocrine syndrome type 1, is a rare autosomal recessive disorder caused by a mutation in the autoimmune regulator (*AIRE*) gene (21q22.3) (1, 2). Patients with APECED typically exhibit the following triad of conditions: hypoparathyroidism, adrenocortical failure, and chronic mucocutaneous candidiasis. Various clinical manifestations and >100 causative mutations have been described (3–6). However, only two cases of autoimmune encephalitis that occurred in patients already diagnosed with APECED have been reported (7, 8), and no previous studies have demonstrated autoimmune encephalitis as an initial manifestation of APECED.

Infantile spasms (IS), frequently referred to as West syndrome (WS), are the most common epileptic encephalopathy that occurs during the first 2 years of life and are characterised by epileptic spasms and hypsarrhythmia on interictal electroencephalography (EEG) (9). While hormonal therapy is currently the main treatment for IS/WS, more effective and aetiology-specific treatments—such as vigabatrin for patients with tuberous sclerosis complex or a ketogenic diet for patients with glucose transporter 1 deficiency—are emerging (10). However, the overall prognosis of patients with IS/WS is poor, with the development of other seizure types, autism, and intellectual disability being common (10). Therefore, in addition to timely diagnosis and treatment, other more effective treatment options are needed.

To our knowledge, no reports of patients with IS/WS and autoimmune encephalitis currently exist, presumably because the median age of paediatric patients with anti-N-methyl-D-aspartate receptor (anti-NMDAR) encephalitis, which is the most frequent in paediatric autoimmune encephalitides, is 13–14 years (11, 12). Furthermore, the youngest patient included in a study thus far was 4 months old, excluding a case of transient anti-NMDAR encephalitis in a newborn infant who experienced transplacental transmission (13, 14). Thus, infants with autoimmune encephalitis including anti-NMDAR encephalitis are rare and the likelihood of such infants developing IS/WS remains unknown (15).

Herein, we describe the patient's response to treatment with rituximab, a chimeric mouse/human monoclonal antibody directed against human CD20, which depletes B cells (16); its efficacy in treating anti-NMDAR encephalitis has deemed it acceptable as a second-line treatment (17). Rituximab treatment unexpectedly resulted in complete cessation of epileptic spasms and resolution of hypsarrhythmia although the efficacy was transient and the patient eventually showed severely delayed developmental milestones. Despite the limited ability to extrapolate findings from a single case, this effect of rituximab treatment suggests that B cells may play a crucial role in IS/WS mechanisms and highlights its potential as a treatment option for IS/WS.

## Case Report

**Figure 1:**
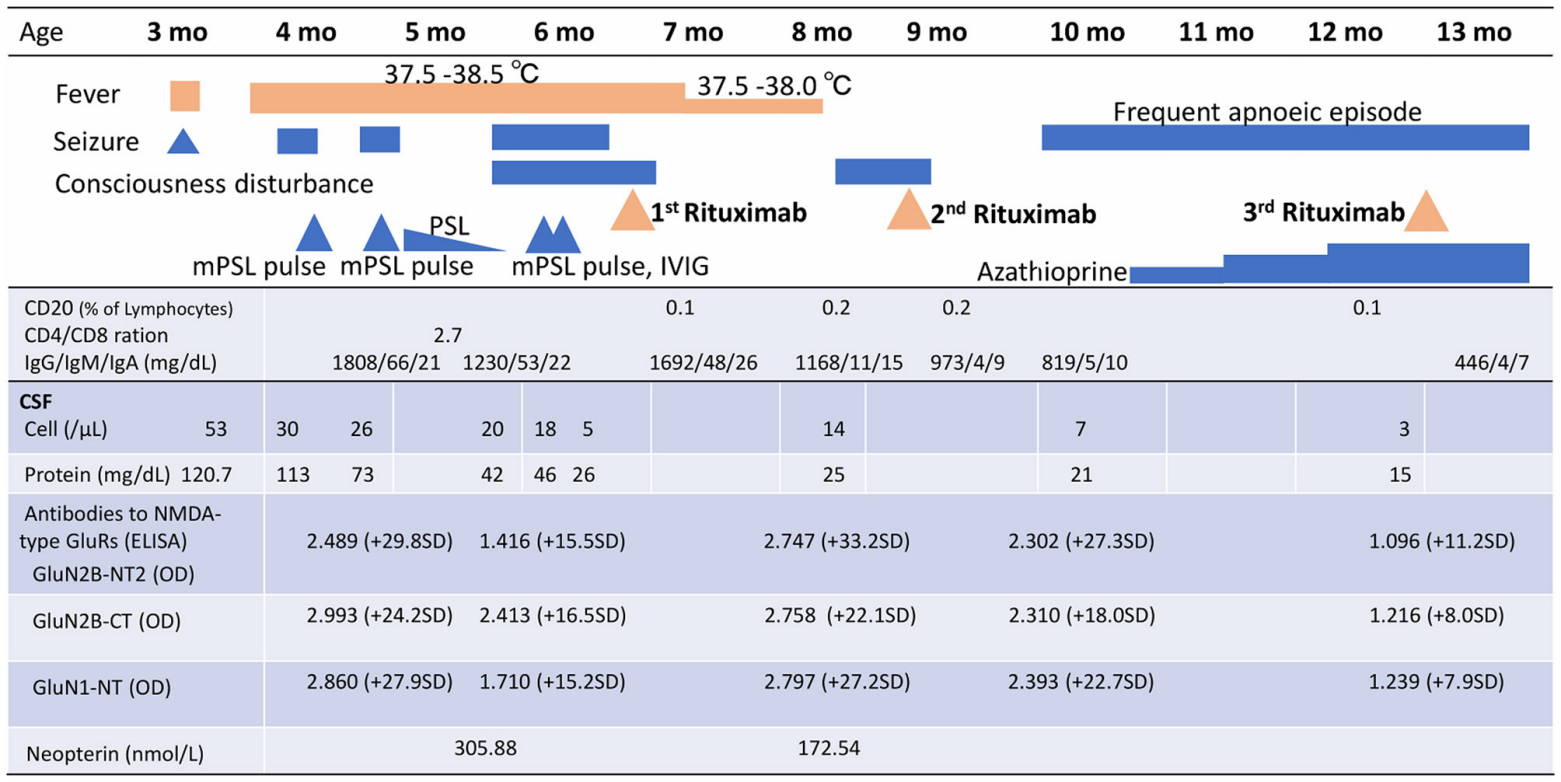
Clinical signs, treatment course, peripheral blood CD20^+^ B cells, and cerebral spinal fluid analysis, including anti-NMDAR antibody titres and neopterin, over time. mPSL, methylprednisolone; PSL, prednisolone; IVIg, intravenous immunoglobulin; CSF, cerebral spinal fluid; NMDA, N-methyl-D-aspartate; GluRs, glutamate receptors; ELISA, enzyme-linked immunosorbent assays; GluN2B-NT2, N-terminal of GluN2B; OD, optical density; GluN2B-CT, C-terminal of GluN2B; GluN1-NT, N-terminal of GluN1; SD, standard deviation.

**Figure 2:**
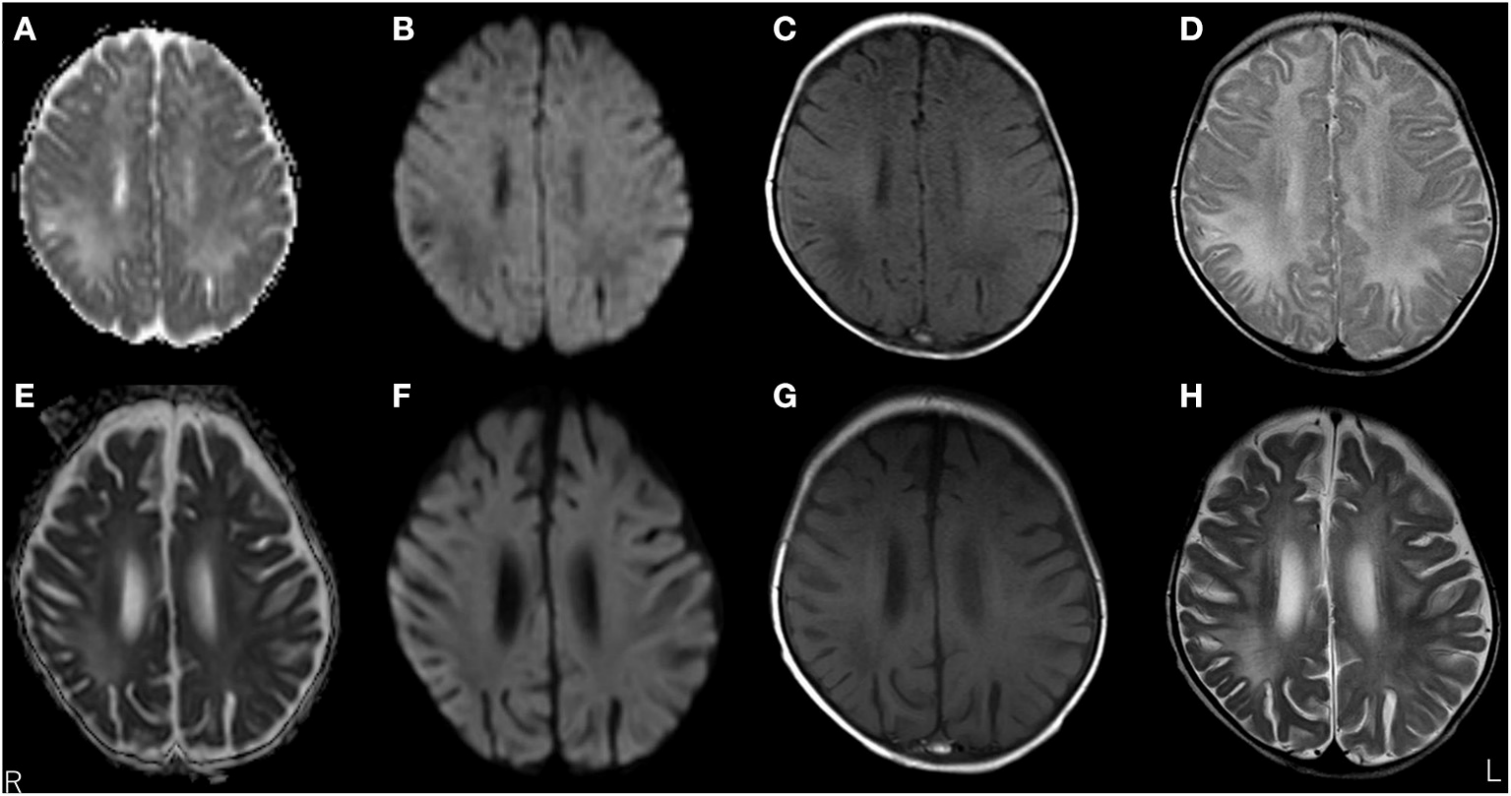
Brain magnetic resonance imaging (MRI) over time. MRI at 3 months old showed white matter lesions with high intensity on apparent diffusion coefficient maps **(A)** and T2 images **(D)**, and low intensity on diffusion-weighted (DW) images **(B)** and T1-weighted images **(C)** within the right frontal and parietal lobes and both occipital lobes, suggesting demyelination. MRI at 5 months old revealed a significant decrease in white matter volume with slight improvements in abnormal lesions within the right frontal, left parietal, and left occipital lobes. No remarkable changes were observed in lesions within the right occipital lobe with high intensity on apparent diffusion coefficient maps **(E)** and T2-weighted images **(H)**, and low intensity on DW images **(F)** and T1-weighted images **(G)**.

A 3-month-old girl was admitted to the referring hospital and presented with a fever and a brief generalised clonic seizure with right deviation in both eyes 10 days after her first hepatitis B vaccination and 3 days after *Haemophilus influenzae* type b vaccination. She was hospitalised in the referring hospital for 1 week with a provisional diagnosis of viral meningitis with elevated white blood cell (53/μL) and protein (120.7 mg/dL) levels in the cerebral spinal fluid (CSF). Two weeks later, she was re-admitted to the hospital with a fever, involuntary movements in all four limbs, and right-side facial palsy. Subsequent CSF examination showed increased white blood cell (30/μL; with 96% lymphocytes) and protein (113 mg/dL) levels (Figure 1), and normal expression of myelin basic protein (<40 pg/mL; normal range: 0–102 pg/mL). Magnetic resonance imaging (MRI) showed white matter lesions suggesting demyelination (Figures 2A–D). The patient was subsequently transferred to our facility.

**Figure 3:**
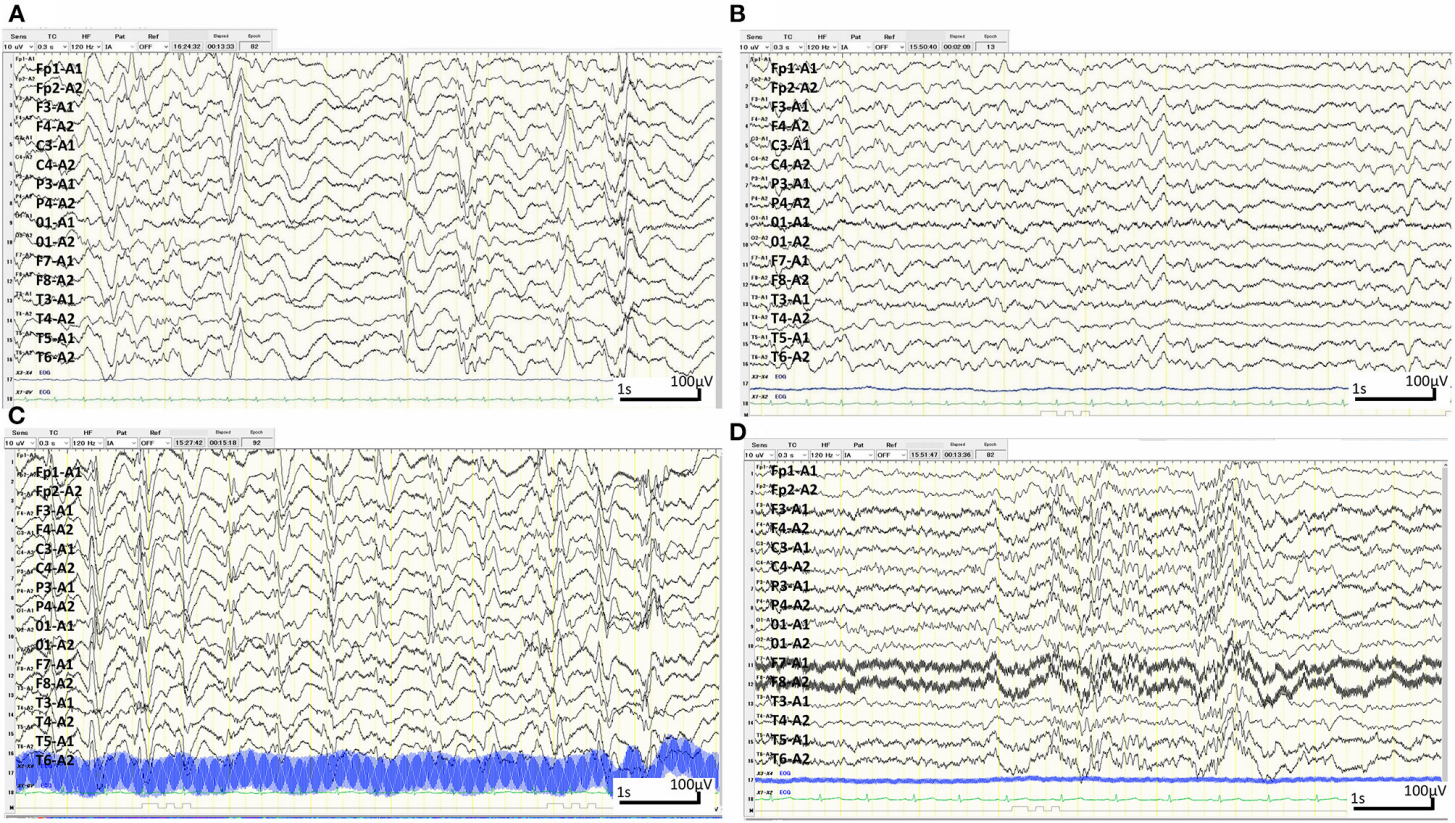
Sleep electroencephalography (EEG) over time. **(A)** Sleep EEG at 6 months old. Interictal EEG showing hypsarrhythmia with multifocal spike- and sharp-wave activity and slow-wave activity exhibiting occasional interhemispheric synchrony and symmetry with recurrent episodes of generalised voltage attenuation. **(B)** Sleep EEG 2 weeks after the first rituximab administration at 7 months of age. Interictal EEG showing complete resolution of hypsarrhythmia and periodic generalised polyspikes, though sharp waves can occasionally be independently observed in the right and left hemispheres. **(C)** Sleep EEG when the patient became lethargic at 8 months of age. Interictal EEG showing a burst of 1.5 Hz slow spike-and-wave activity. **(D)** Sleep EEG at 12 months of age. EEG showing bursts of generalised fast activity (10–25 Hz) without clinical seizures.

Upon admission to our hospital, the patient's interictal EEG showed no abnormalities. All routine blood investigations were normal except for decreased complement component 4 (3 mg/dL) and 50% haemolytic complement activity (12.0 CH_50_/mL). The CSF oligoclonal band test was positive, and myelin basic protein expression was negative. Serum immunoglobulin (Ig)A and IgM levels were within normal ranges, but the IgG level was elevated (1,808 mg/dL). Possible herpes simplex virus infection was ruled out by polymerase chain reaction assessment of the CSF and consecutive serum samples. The patient's parents are not in a consanguineous marriage and both are healthy. Acute disseminated encephalomyelitis or autoimmune encephalitis was suspected at this point, and methylprednisolone (mPSL) pulse therapy was initiated. Two courses of intravenous mPSL pulse therapy (30 mg/kg/dose for 3 days) and prednisolone (started at 2 mg/kg/day) with tapering doses later improved the facial palsy and decreased the seizure frequency. The interictal EEG showed frequent spikes and slow waves in the right hemisphere at 5 months of age and was accompanied by a persistent fluctuating fever (37.5–38.5°C). MRI at 5 months of age revealed a significant decrease in the volume of white matter (Figures 2E–H). After completing a course of prednisolone administration, the patient's frequent seizures returned despite the administration of multiple antiepileptic medications. She developed IS/WS at 6 months of age, showing the typical EEG findings (Figure 3A). Sudden flexions of both lower extremities frequently occurred in clusters. She was also diagnosed with diabetes mellitus type 1 due to hyperglycaemia (random venous plasma glucose ≥ 200 mg/dL, up to 420 mg/dL) with a low urine C peptide level (5.9 μg/day) at 6 months of age, requiring continuous infusion of insulin, which was later switched to subcutaneous insulin injections. The neopterin level in the CSF, which acts as a non-specific biomarker of central nervous system inflammation (18), was markedly elevated (normal range for 1–12-month-old infants: 10.3–34.6 nmol/L; Figure 1). After the third mPSL pulse therapy and a single dose of intravenous Ig (1 g/kg) were administered without significant effects on the clinical course, the results of anti-NMDAR antibody titres using a cell-based assay and antibodies to NMDA-type glutamate receptors (GluRs) examined using conventional enzyme-linked immunosorbent assays in CSF at 4 months of age were positive (Figure 1) (19, 20). These results led to the diagnosis of autoimmune encephalitis and initiation of rituximab treatment.

After obtaining hospital approval and parental consent, the first dose of rituximab (375 mg/body surface area, m^2^) was administered at 6 months of age with one dose of antihistamine (hydroxyzine hydrochloride 1 mg/kg/dose) and corticosteroid administration (mPSL 1 mg/kg/dose) without adverse events. Trimethoprim-sulfamethoxazole (Trimethoprim 5 mg/kg/day) was administered every other day for infection prophylaxis. The patient's consciousness level improved and her frequent epileptic spasms completely stopped several days after the first rituximab administration. The patient's fever gradually decreased from 37.5–38.5°C to 37.5–38.0°C. The EEG results 2 weeks after rituximab administration also improved dramatically (Figure 3B). Complete resolution of hypsarrhythmia and periodic generalised polyspikes were also confirmed through sleep EEG at 3, 4, and 5 weeks after the first rituximab administration, although occasional spikes were observed in the right frontal and central regions. Glycated haemoglobin (HbA1c) was 7.3% (≥6.5% using an assay that is certified by the National Glycohaemoglobin Standardisation Program) at 7 months of age. Neopterin in the CSF was reduced, while antibodies to NMDA-type GluRs titres in the CSF further increased at 8 months of age (Figure 1).

The patient's consciousness deteriorated at 8 months of age and she remained somnolent. EEG revealed generalised 1.5 Hz slow spike-and-wave activity during sleep (Figure 3C), while continuous video recording EEG revealed no clinical seizures. After a second rituximab administration at 8 months of age, she began to babble and turn her head towards sounds, follow moving objects with her eyes from side to side, and exhibit increased voluntary and smooth movements with her arms and legs. When the patient was 6 months old, her 2-year-old sister developed autoimmune hepatitis and retinopathy and was admitted to another hospital (21). Genetic testing was performed in both our patient and her sister, revealing a known pathogenic homozygous variant in the *AIRE* gene (c.415C>T, p.Arg139^*^) (22), resulting in the diagnosis of APECED when our patient was 10 months old.

The patient started having frequent brief apnoeic episodes during both wakefulness and sleep at 10 months of age, which was not accompanied by EEG abnormalities, as determined via continuous video EEG monitoring, or any problems in the upper and lower airways, as revealed via computed tomography and endoscopic examinations conducted by an otolaryngologist. After the genotypes of NUDT15 p.Arg139 were checked and no Arg139Cys variant was identified in the patient, because of its strong association with adverse events derived from thiopurines, such as leukopenia and alopecia in Asians (23), azathioprine administration was initiated at 1 mg/kg/day and increased to 2.5 mg/kg/day. A third rituximab course was administered at 12 months of age without significant changes in the frequency of apnoeic episodes. Eventually, the patient required non-invasive positive pressure ventilation during night-time for central alveolar hypoventilation. Although she has not had any clinical seizures since 7 months of age, she showed severely delayed neurodevelopmental milestones (3 months old at her chronological age of 14 months) along with abnormal EEG findings (Figure 3D). She has not had any episodes of candida infection, including mucocutaneous candidiasis, or any clinical signs of hypoparathyroidism, or adrenocortical failure. Recurrent examinations of her thyroid, parathyroid, and adrenal hormone levels revealed no abnormalities. Serum glutamic acid decarboxylase, anti-insulinoma-associated protein 2, and anti-insulin antibodies were examined multiple times (at 6, 7, and 9 months of age) and were all negative. Furthermore, she has not experienced any episodes of serious infection with trimethoprim-sulfamethoxazole (Trimethoprim 5 mg/kg/day) administered every other day. She has not had any adverse events, such as leukopenia, alopecia, or liver dysfunction, related with azathioprine administration although IVIG administration (500 mg/kg/dose) was needed at 14 months of age for hypogammaglobulinaemia (446 mg/dL) due to rituximab administrations.

## Discussion

We encountered an infant with IS/WS and anti-NMDAR encephalitis associated with APECED. The following three important findings were identified: (1) autoimmune encephalitis can present as an initial manifestation of APECED; (2) autoimmune encephalitis can cause infants to develop IS/WS; and (3) rituximab was able to completely cease the epileptic spasms and resolve the hypsarrhythmia, although the efficacy for encephalitis was transient and repeated administration was required.

APECED can initially present as autoimmune encephalitis. Before the appearance of the second condition of the triad, 23% of patients reportedly have other symptoms, including chronic diarrhoea, keratitis, periodic rash with fever, severe constipation, autoimmune hepatitis, alopecia, enamel hypoplasia, and vitiligo (3, 4, 6). The patient's diagnosis of autoimmune encephalitis and diabetes mellitus at a young age and her older sister's concurrent development of autoimmune hepatitis led to a genetic analysis that resulted in the diagnosis of APECED. *AIRE* is expressed in thymic medullary epithelial cells and promotes the expression of tissue-specific antigens, which allow autoreactive T cells with an affinity for self-proteins to either die by apoptosis or become forkhead box P3 (FoxP3)-expressing regulatory T cells (central T-cell tolerance). The induction of FoxP3-expressing regulatory T cells in the thymus limits the activities of autoreactive cells (peripheral T-cell tolerance). When *AIRE* is lacking, many autoreactive T cells can escape into the general circulation. A lack of FoxP3-expressing regulatory T cells also halts the inhibition of autoreactive T cells (5).

To date, this is the first case report of a patient with autoimmune encephalopathy who developed IS/WS. However, it is important to note that a 4-month-old girl with voltage-gated potassium channel-complex was reported to show encephalopathy and early-onset epileptic spasms, without an obvious encephalitic illness (24). The incidence of autoimmune encephalitis during early life periods is quite low. However, a study screening for neuronic autoantibodies in paediatric patients with epileptic encephalopathies—64% of whom were diagnosed with IS/WS and/or Lenox–Gastaut syndrome of an unknown cause—revealed that 14% (7/50 patients) were positive for neuronic antibodies many years after onset, including two patients positive for NMDAR antibodies (25). Our case strongly suggests that anti-NMDAR antibodies cause IS/WS. Pathological activation of a network involving cortical and subcortical structures can lead to the development of IS/WS, and cerebral cortex lesions without specific locations are usually found in patients with IS/WS (26, 27). Continuous inflammation may also lead to a decrease in the seizure threshold, thus contributing to the development of IS/WS.

In our case, rituximab treatment unexpectedly resulted in complete cessation of epileptic spasms and resolution of hypsarrhythmia. Rituximab is a monoclonal antibody against human CD20 that depletes B cells and a small fraction of plasma cells and is currently considered a second-line treatment for anti-NMDAR encephalitis (16, 17). Rituximab cannot deplete most plasma cells that produce IgG. Thus, while a low-grade fever of 37.5–38.0°C persisted for 1 month after the first rituximab administration, the frequent spam cessation and EEG improvement several days following the first rituximab treatment suggest that the drug directly improved IS/WS through interactions between B and T cells rather than indirectly through anti-NMDAR antibodies. Furthermore, our patient did not experience any adverse events due to rituximab administrations, except that she required IVIG administration for hypogammaglobulinaemia at 14 months of age. Despite the limited ability to extrapolate findings from a single case, rituximab's effects may suggest that B cells play a crucial role in IS/WS mechanisms. Rituximab was administered multiple times safely to our patient with WS and Anti-NMDAR Encephalitis Associated with APECED as previously reported in paediatric patients (28). Therefore, rituximab should be investigated to determine whether it is an aetiology-specific treatment suitable only for IS/WS patients with autoimmune encephalitis or whether it is effective for IS/WS patients with other underlying mechanisms.

Neuroinflammation suppression may represent one factor in the underlying mechanism of adrenocorticotropic hormone therapy for IS/WS (26) besides corticotropin-releasing hormone suppression, which may actually induce convulsions in the immature brain (29, 30). The interferon-γ level, number of CD4^+^ cells, and CD4^+^/8^+^ ratio were higher before adrenocorticotropic hormone therapy than after in patients with IS/WS (31, 32). Interleukin-6 and interleukin-17 levels were also significantly higher in untreated patients with IS/WS than in healthy controls (33). In addition, paradoxical spontaneous IS/WS remission has been reported after viral infections (34). These previous studies suggest that interactions between B and T cells play a role in the mechanisms underlying IS/WS, and that rituximab or T-cell suppressant medications, such as tacrolimus, may represent a future treatment option for IS/WS because B cells not only produce antibodies but also regulate dendritic cells and T-cell subset functions through cytokine production (35).

In conclusion, despite the limited ability to extrapolate findings from a single case, the favourable treatment response to rituximab in a rare case of an infant with IS/WS, may suggest that B and T cells play a crucial role in the underlying IS/WS mechanisms. Further, rituximab is also worth investigating to clarify whether it is an aetiology-specific treatment for IS/WS patients with autoimmune encephalitis, or whether it is effective for IS/WS patients with other underlying pathologies.

## Data Availability Statement

The original contributions presented in the study are included in the article/supplementary material, further inquiries can be directed to the corresponding author/s.

## Ethics Statement

The studies involving human participants were reviewed and approved by the institutional review board of St Mary's Hospital, Fukuoka, Japan (IRB number: 20-0105). Written informed consent to participate in this study was provided by the participants' legal guardian/next of kin. Informed consent for publication was obtained from both parents.

## Author Contributions

GK, RN, YT, and TM contributed to the study design, data curation, interpretation of the results, and manuscript writing. GK, YT, KO, RN, YT, HS, and TM contributed to the research. All authors have reviewed and approved the final version of this manuscript.

## Conflict of Interest

The authors declare that the research was conducted in the absence of any commercial or financial relationships that could be construed as a potential conflict of interest.

## Abbreviations

APECED: autoimmune polyendocrinopathy-candidiasis-ectodermal dystrophy
AIRE: autoimmune regulator
CSF: cerebral spinal fluid
EEG: electroencephalography
FoxP3: forkhead box P3
GluRs: glutamate receptors
Ig: immunoglobulin
IS: infantile spasms
mPSL: methylprednisolone
MRI: magnetic resonance imaging
NMDAR: N-methyl-D-aspartate receptor
WS: West syndrome.
